# Beyond Surgical Follow-Up: Measuring the Long-Term Burden of Permanent Hypoparathyroidism After Thyroid Cancer Surgery

**DOI:** 10.3390/medsci14060650

**Published:** 2026-10-09

**Authors:** Rossella Melcarne, Jose Luis Carrillo Lizarazo, Marco Biffoni, Laura Giacomelli, Gianluca Donatini, Cosimo Durante, Giorgio Grani

**Affiliations:** 1 Department of General and Specialized Surgery and Anesthesiology, Sapienza University of Rome, 00185 Rome, Italy; marco.biffoni@uniroma1.it (M.B.); laura.giacomelli@uniroma1.it (L.G.); 2 Department of General and Endocrine Surgery, Centre Hospitalier Universitaire (CHU) de Poitiers, 86000 Poitiers, France; luiscarrillo849@gmail.com (J.L.C.L.); gianluca.donatini76@gmail.com (G.D.); 3 Department of Translational and Precision Medicine, Sapienza University of Rome, 00185 Rome, Italy; cosimo.durante@uniroma1.it (C.D.); giorgio.grani@uniroma1.it (G.G.)

**Keywords:** differentiated thyroid cancer, hypoparathyroidism, thyroidectomy, central neck dissection, surgical complications, quality of care, risk stratification

## Abstract

**Background:** Permanent postoperative hypoparathyroidism (p-HypoPT) is a major long-term complication of thyroid surgery; however, its incidence remains uncertain because reported rates are influenced by heterogeneous case mix and variable follow-up. We assessed the burden and determinants of p-HypoPT in patients undergoing surgery for differentiated thyroid cancer (DTC) with long-term endocrine follow-up. **Methods:** We retrospectively analysed prospectively collected data from 518 consecutive adults with DTC enrolled in a dedicated longitudinal endocrine follow-up programme between 2013 and 2022. p-HypoPT was operationally defined as the persistent requirement for oral calcium and/or active vitamin D supplementation at ≥12 months after surgery. Independent associations were assessed by means of multivariable logistic regression. Rates were also explored across the 2015 and 2025 American Thyroid Association (ATA) risk categories. **Results:** p-HypoPT occurred in 60/518 patients (11.6%). Median follow-up was 10.8 years (IQR 8.0–12.8), and all patients with p-HypoPT remained on calcium and/or active vitamin D supplementation at their latest available follow-up. Central neck dissection was associated with p-HypoPT in multivariable analysis (adjusted OR 2.94, 95% CI 1.55–5.57; *p* < 0.001). Under the 2025 ATA classification, p-HypoPT frequencies differed across categories in unadjusted analysis (*p* = 0.005), with the highest frequency in the high-intermediate-risk group (23.9%). This association was attenuated after adjustment for central and lateral neck dissection and was no longer statistically significant (global *p* = 0.566). **Conclusions:** Permanent hypoparathyroidism represents a relevant long-term burden after DTC surgery. Its assessment should account for both the duration of postoperative surveillance and the extent of surgery, moving beyond aggregate rates derived from heterogeneous thyroidectomy cohorts. More granular, procedure-specific and risk-adjusted estimates may ultimately support meaningful quality comparisons and provide a more individualised assessment of long-term functional risk.

## 1. Introduction

As survival after differentiated thyroid cancer (DTC) continues to improve, long-term functional outcomes have become central measures of quality of care [1,2]. Their accurate assessment depends not only on standardised definitions but also on the duration and setting of follow-up [3].

Permanent postoperative hypoparathyroidism (p-HypoPT) exemplifies this challenge. It is the most common long-term complication of total thyroidectomy and the leading cause of chronic hypoparathyroidism worldwide [3,4]. Although DTC carries an excellent prognosis [5], successful surgery may result in a lifelong endocrine disorder requiring chronic treatment and long-term surveillance, making p-HypoPT a clinically relevant long-term functional outcome and an increasingly recognised component of quality assessment in thyroid surgery [6,7].

Nevertheless, reported frequencies vary widely. Most surgical series report p-HypoPT rates between 1% and 4%, whereas real-world studies and endocrine registries consistently describe substantially higher frequencies [8,9,10]. Importantly, many of these estimates are derived from heterogeneous surgical cohorts combining patients operated on for malignant and benign thyroid diseases, often with markedly different disease complexity and extent of surgery. Consequently, overall rates may largely reflect the underlying case mix and provide limited information on the reported frequencies of p-HypoPT within specific clinical settings, such as differentiated thyroid cancer, multinodular goitre, or other indications for total thyroidectomy. This heterogeneity, combined with differences in definitions and follow-up strategies, makes direct comparison across studies difficult and leaves the indication-specific burden of permanent hypoparathyroidism incompletely defined [11].

**Figure 1:**
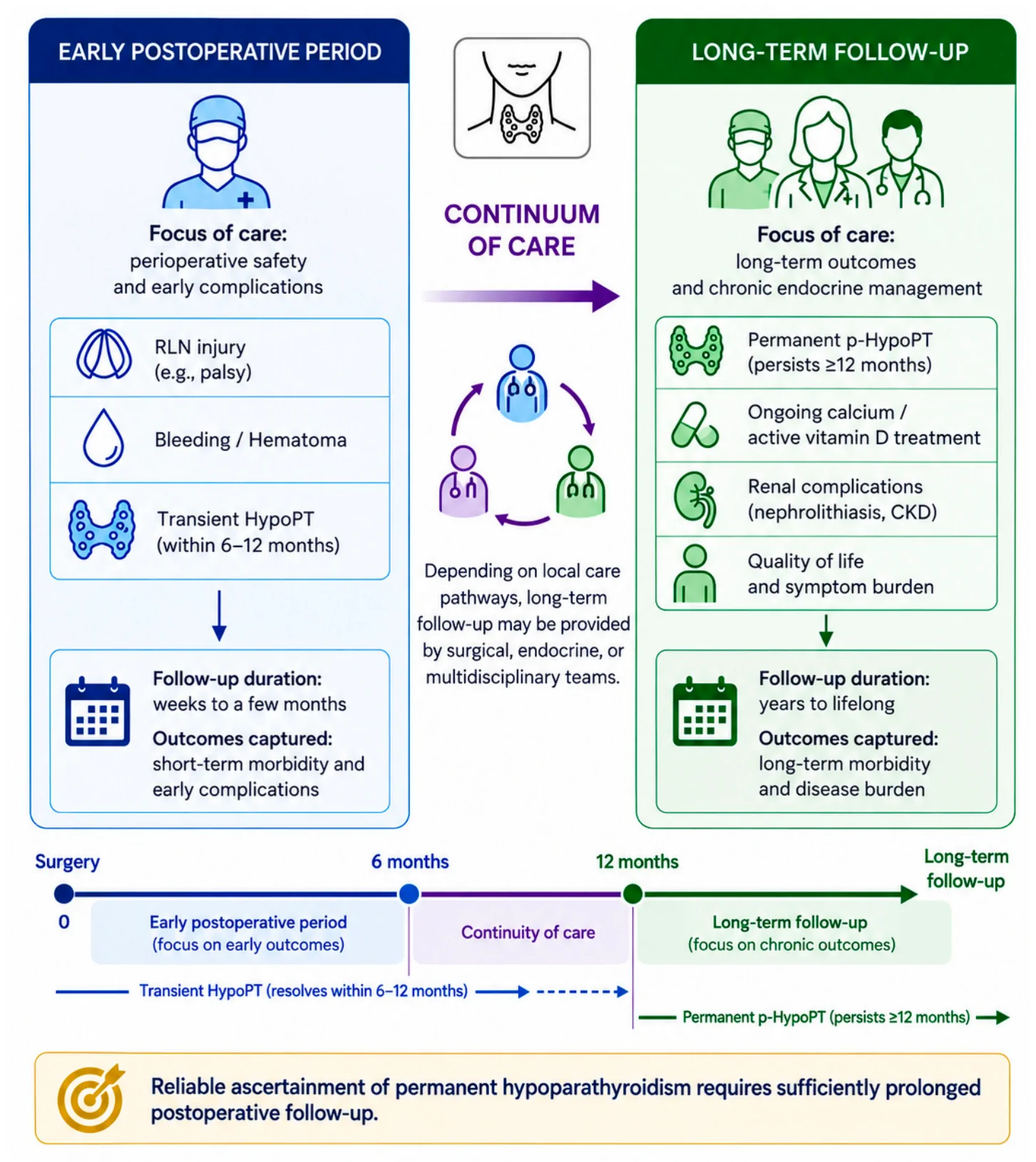
**Continuum of postoperative care after thyroid cancer surgery and ascertainment of permanent hypoparathyroidism.** *Conceptual schematic illustrating the temporal continuum of care after thyroid cancer surgery. Assessment of persistent parathyroid dysfunction requires long-term surveillance extending beyond the early postoperative period. Depending on local care pathways, this follow-up may be provided by surgical, endocrine, or multidisciplinary teams. Abbreviations: HypoPT, hypoparathyroidism; RLN, recurrent laryngeal nerve; CKD, chronic kidney disease. Figure 1 was created with the assistance of ChatGPT (GPT-5.5 Instant, OpenAI; image-generation functionality, accessed in June 2026; exact model version not retained). The AI tool was used only to support the graphical rendering of an author-defined conceptual framework. The scientific concept, structure, labels, and interpretation presented in the figure were developed, reviewed, and approved by the authors*.

A distinctive feature of p-HypoPT is the temporal disconnect between surgical injury and diagnosis. According to the 2022 International Task Force guidelines, this permanent disease can only be diagnosed after persistence beyond 12 months [3,4]. By that time, patients have often transitioned from surgical follow-up to endocrine care. Consequently, the reported frequency of p-HypoPT may depend not only on surgical quality but also on methods, setting, and duration of outcome assessment (Figure 1).

This temporal disconnect has important methodological implications. Surgical registries primarily capture perioperative events and early complications, whereas longitudinal endocrine assessment documents the long-term evolution of calcium homeostasis and chronic replacement therapy.

Accurate quantification of this burden is relevant beyond epidemiology. It may improve preoperative shared decision-making, facilitate identification of patients at highest risk, and support more comprehensive evaluation of long-term treatment outcome [5,6,12,13].

To address these issues, we analysed a consecutive cohort of patients with DTC enrolled in a dedicated longitudinal follow-up programme. Our objectives were to quantify the observed frequency and long-term burden of p-HypoPT, identify clinicopathological and surgical factors associated with its occurrence, and explore the relationship between contemporary ATA risk stratification and long-term functional morbidity following thyroid cancer surgery.

## 2. Methods

### 2.1. Study Design and Population

In this retrospective observational study, we analysed prospectively collected data from consecutive adult patients (≥18 years) with histologically confirmed differentiated thyroid cancer (DTC) who entered a dedicated endocrine follow-up pathway between January 2013 and January 2022 at the Translational and Precision Medicine Unit, AOU Policlinico Umberto I, Sapienza University of Rome. Patients had undergone thyroid surgery at different hospitals within the Rome metropolitan area and were subsequently followed by the same specialist endocrine team for long-term thyroid cancer surveillance. Follow-up consisted of structured annual reassessment irrespective of disease severity, oncological status, or postoperative complications and included clinical evaluation, biochemical monitoring, and adjustment of ongoing endocrine therapy. Data were prospectively collected within the longitudinal follow-up programme and retrospectively analysed for the purposes of the present study. Data were extracted in June 2025.

Patients were identified from a prospectively maintained endocrine follow-up database containing demographic, clinical, biochemical, pathological, treatment, and longitudinal follow-up data. Eligible patients were required to have at least 12 months of postoperative observation. Patients with pre-existing hypoparathyroidism, previous neck surgery, chronic calcium supplementation for indications unrelated to postoperative parathyroid dysfunction, incomplete clinical records, or insufficient information to ascertain permanent postoperative hypoparathyroidism were excluded.

During endocrine follow-up, calcium and active vitamin D supplementation were periodically reassessed based on clinical status and serum calcium and phosphate levels. When clinically appropriate, treatment was progressively reduced to assess ongoing supplementation requirements. A formal protocol mandating complete withdrawal of supplementation was not systematically applied.

The study is reported in accordance with the Strengthening the Reporting of Observational Studies in Epidemiology (STROBE) recommendations (Supplementary File S1).

### 2.2. Data Collection

Demographic variables included age and sex. Clinicopathological variables comprised histological subtype, primary tumour size, extrathyroidal extension, lymph node involvement, surgical margins, and radioactive iodine treatment.

Surgical variables included the extent of thyroidectomy and the performance of central and lateral neck dissection. The indication for central neck dissection (prophylactic vs. therapeutic) and its laterality were not consistently available across centres and were therefore not included in the analysis.

Recurrence risk was classified according to the 2015 American Thyroid Association (ATA) Risk Stratification System [14], which represented the standard of care during the study period. Response to therapy at 12 months was also classified according to the 2015 ATA dynamic risk stratification criteria.

To explore whether the updated oncological classification also reflected long-term functional morbidity, all patients were subsequently reclassified according to the 2025 ATA Risk Stratification System [5], and the frequency of permanent postoperative hypoparathyroidism was compared across the original three-tier and updated four-tier classification models.

### 2.3. Outcome Measures

The primary outcome was permanent postoperative hypoparathyroidism (p-HypoPT), operationally defined as the persistent requirement for oral calcium and/or active vitamin D supplementation at ≥12 months after surgery. The specific long-term replacement regimen and doses of calcium and active vitamin D were not systematically captured in the study database and were therefore not analysed. Serum calcium and phosphate were available during long-term endocrine follow-up, whereas PTH measurements were not systematically available in the study database and were therefore not required for outcome classification. Patients receiving chronic calcium supplementation for indications unrelated to postoperative parathyroid dysfunction were excluded. During endocrine follow-up, supplementation was progressively reduced whenever clinically appropriate to assess residual parathyroid function; however, a standardised protocol requiring complete treatment withdrawal was not routinely applied.

Through secondary analyses, we aimed to identify clinicopathological and surgical factors associated with the development of p-HypoPT and to evaluate whether ATA risk stratification was associated with long-term functional morbidity.

### 2.4. Statistical Analysis

Continuous variables are presented as mean ± standard deviation (SD) or median with interquartile range (IQR), as appropriate. Categorical variables are reported as frequencies and percentages.

Baseline characteristics were compared using Pearson’s χ^2^ test or Fisher’s exact test for categorical variables, Welch’s t-test for age, and the Mann–Whitney U test for continuous tumour size.

Variables considered clinically relevant or associated with p-HypoPT in univariable analyses (*p* < 0.10) were entered into a multivariable logistic regression model to assess factors independently associated with permanent postoperative hypoparathyroidism. Multivariable analyses were performed using complete cases, with no imputation of missing values. Multicollinearity was assessed using variance inflation factors. Model discrimination was evaluated using the area under the receiver-operating-characteristic curve, and calibration using the Hosmer–Lemeshow test and Brier score. A parsimonious sensitivity model was also fitted using variables showing the strongest univariable association with p-HypoPT. Given the substantial proportion of unknown pathological nodal status, two sensitivity analyses were performed: one treating unknown nodal status as a separate category and one restricted to patients with documented nodal status. Results are reported as odds ratios (ORs) with 95% confidence intervals (95% CIs).

A hypothesis-generating analysis was subsequently performed after retrospective reclassification according to the 2025 ATA Risk Stratification System [5]. The distribution of p-HypoPT across ATA 2015 and ATA 2025 categories was assessed using categorical comparisons, and unadjusted odds ratios (ORs) with 95% confidence intervals (95% CIs) were calculated using the low-risk category as reference. Separate multivariable logistic regression models were fitted for the ATA 2015 and ATA 2025 classifications, each including ATA risk category, central neck dissection, and lateral neck dissection. The overall contribution of ATA category was assessed using a likelihood-ratio test. A sensitivity analysis was additionally performed for the ATA 2025 model after restriction to patients undergoing total thyroidectomy.

All statistical analyses were performed using JASP software (version 0.18.1). All tests were two-sided, and a *p* value < 0.05 was considered statistically significant.

### 2.5. Ethics

The study was conducted within the framework of the Italian Thyroid Cancer Observatory (ITCO) multicentre observational study (ClinicalTrials.gov identifier: NCT04031339). The current study protocol and data-collection framework were approved by the Territorial Ethics Committee Lazio Area 1 (Ref. 3366; Protocol No. 0707/2024; substantial Amendment No. 5 approved 31 July 2024). Written informed consent for the collection and scientific use of anonymised clinical data was obtained from all participants.

### 2.6. Use of Generative Artificial Intelligence Tools

Generative artificial intelligence tools were used solely to support language refinement, the drafting of selected portions of the manuscript, and the preparation of selected figures. All AI-assisted content and visual material were critically reviewed, revised, and approved by the authors, who take full responsibility for the final manuscript.

## 3. Results

### 3.1. Study Population

Of the 736 patients identified in the source endocrine follow-up database, 214 were excluded because the primary outcome could not be ascertained with sufficient certainty from the available records. The remaining 522 patients had an adequately documented p-HypoPT status and were included in the original analytic cohort. Following data re-audit, four Follicular Neoplasm with Uncertain Malignant Potential (FT-UMP) cases were subsequently excluded because they did not meet the revised histological eligibility criteria, resulting in a final analytic cohort of 518 patients (Supplementary Figure S1; Supplementary Table S1). At database extraction in June 2025, median interval from thyroid surgery to the latest available follow-up was 10.8 years (IQR 8.0–12.8). All patients had more than 24 months of follow-up, and 510/518 (98.5%) had been followed for more than 5 years. The study population comprised 371 women (71.6%) and 147 men (28.4%), with a mean age of 48.1 years. Most patients underwent total thyroidectomy (494/518, 95.4%), whereas lobectomy was performed in 24/518 (4.6%). According to the 2015 ATA risk stratification system, 317 patients (61.2%) were classified as low risk, 180 (34.7%) as intermediate risk, and 21 (4.1%) as high risk. Baseline demographic, pathological, and treatment characteristics are summarised in Table 1.

### 3.2. Clinical Characteristics According to Permanent Postoperative Hypoparathyroidism

Permanent postoperative hypoparathyroidism occurred in 60 of 518 patients (11.6%). Among the 60 patients with p-HypoPT, median follow-up was 9.0 years (IQR 7.4–11.6), and all remained on calcium and/or active vitamin D supplementation at their latest available follow-up. Clinical characteristics according to parathyroid status are summarised in Table 2. Among the 494 patients who underwent total thyroidectomy, central neck dissection status was known in 493: 342 underwent total thyroidectomy alone, whereas 151 underwent total thyroidectomy with central neck dissection. The corresponding p-HypoPT rates were 7.6% (26/342; 95% CI 5.2–10.9) and 22.5% (34/151; 95% CI 16.6–29.8), respectively. Among procedures including lateral neck dissection, p-HypoPT occurred in 13/47 patients (27.7%, 95% CI 16.9–41.8). No statistically significant differences were observed in age, sex, histological subtype, primary tumour size, extrathyroidal extension, surgical margins, radioactive iodine treatment, or response to therapy at 12 months.

### 3.3. Factors Associated with Permanent Postoperative Hypoparathyroidism

Univariable and multivariable logistic regression analyses are presented in Table 3.

In the univariable analysis, pathological nodal involvement, central neck dissection, and lateral neck dissection were significantly associated with permanent postoperative hypoparathyroidism. Compared with patients without nodal metastases, those with lateral compartment involvement had higher odds of p-HypoPT (OR 3.38, 95% CI 1.57–7.28; *p* = 0.002), whereas central compartment involvement alone was not significantly associated with the outcome. Central neck dissection (OR 3.67, 95% CI 2.12–6.37; *p* < 0.001) and lateral neck dissection (OR 3.44, 95% CI 1.70–6.98; *p* < 0.001) were also associated with increased odds of p-HypoPT.

In the multivariable model, central neck dissection remained independently associated with p-HypoPT (adjusted OR 2.94, 95% CI 1.55–5.57; *p* < 0.001). Female sex (adjusted OR 1.93, 95% CI 0.96–3.88; *p* = 0.067), primary tumour size (adjusted OR 1.23 per 10-mm increase, 95% CI 0.95–1.59; *p* = 0.115), extrathyroidal extension, and lateral neck dissection were not independently associated with the outcome.

Sensitivity analyses addressing the high proportion of unknown pathological nodal status yielded consistent results. Central neck dissection remained associated with p-HypoPT when unknown nodal status was modelled as a separate category (adjusted OR 2.31, 95% CI 1.10–4.84; *p* = 0.027) and when the analysis was restricted to patients with documented nodal status (adjusted OR 2.54, 95% CI 1.17–5.53; *p* = 0.018).

### 3.4. Permanent Postoperative Hypoparathyroidism According to ATA Risk Stratification

**Figure 2:**
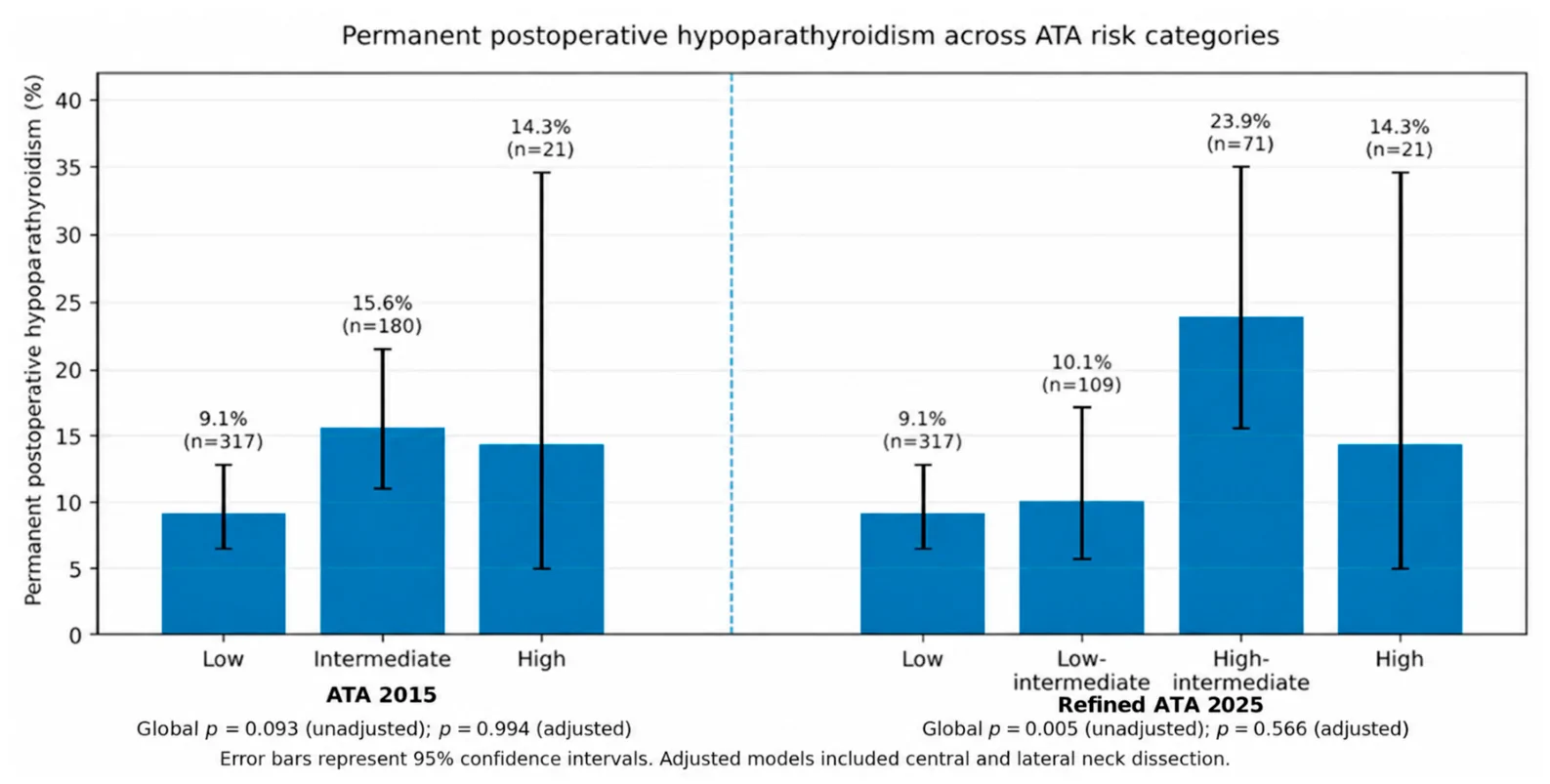
**Permanent postoperative hypoparathyroidism across ATA 2015 and refined ATA 2025 risk categories.** *Bars represent the proportion of patients with permanent postoperative hypoparathyroidism (p-HypoPT) within each risk category; error bars represent 95% confidence intervals. Global unadjusted comparisons were performed using Pearson’s χ^2^ test. Adjusted analyses included central and lateral neck dissection. ATA, American Thyroid Association*.

Under the ATA 2015 classification, p-HypoPT occurred in 29/317 low-risk patients (9.1%), 28/180 intermediate-risk patients (15.6%), and 3/21 high-risk patients (14.3%), with no statistically significant overall difference across categories (*p* = 0.093) (Figure 2).

Under the ATA 2025 classification, p-HypoPT occurred in 29/317 low-risk patients (9.1%, 95% CI 6.4–12.8), 11/109 low–intermediate-risk patients (10.1%, 95% CI 5.7–17.2), 17/71 high–intermediate-risk patients (23.9%, 95% CI 15.5–35.0), and 3/21 high-risk patients (14.3%, 95% CI 5.0–34.6). The overall unadjusted distribution differed across categories (*p* = 0.005). Compared with the low-risk group, the high-intermediate-risk group showed higher odds of p-HypoPT (OR 3.13, 95% CI 1.61–6.08; *p* < 0.001), whereas no significant differences were observed for the low-intermediate- or high-risk groups (Table 4; Figure 2).

After adjustment for central and lateral neck dissection, the overall association between ATA 2025 category and p-HypoPT was attenuated and was no longer statistically significant (global *p* = 0.566). None of the individual ATA categories remained significantly associated with p-HypoPT, whereas central neck dissection remained associated with the outcome (adjusted OR 3.11, 95% CI 1.65–5.88; *p* < 0.001). A sensitivity analysis restricted to patients undergoing total thyroidectomy yielded consistent findings (global adjusted *p* = 0.468) (Table 4).

## 4. Discussion

In the present study, we provide a long-term assessment of permanent postoperative hypoparathyroidism (p-HypoPT) in patients with differentiated thyroid cancer followed within a structured longitudinal surveillance programme. Three main findings emerge. First, p-HypoPT affected approximately one in ten patients in this cohort, confirming that persistent parathyroid dysfunction represents a clinically relevant long-term consequence of thyroid cancer treatment. Second, central neck dissection remained independently associated with p-HypoPT within the available multivariable model, whereas most tumour-related characteristics were not. Third, ATA 2025 categories were associated with different unadjusted frequencies of p-HypoPT, particularly within the high–intermediate-risk group; however, this association was attenuated after adjustment for surgical extent, suggesting a close relationship between oncological burden, surgical complexity, and long-term functional morbidity rather than an independent predictive role of ATA classification [15,16,17,18].

The clinical relevance of the present findings lies not only in the observed frequency of p-HypoPT but also in what they reveal about the challenges of capturing long-term functional morbidity after thyroid cancer surgery. Every case of p-HypoPT represents a patient who, despite successful treatment of a malignancy with an excellent prognosis, develops a chronic endocrine disorder requiring long-term, and potentially lifelong, medical care. In an era in which most differentiated thyroid cancers are highly curable, preservation of long-term endocrine function should be regarded as an integral component of surgical success rather than a secondary outcome [12,19,20]. Accurate characterisation of this burden is therefore relevant not only from an epidemiological perspective but also for healthcare planning, resource allocation, and the evaluation of interventions aimed at reducing this complication [21,22].

The increasing availability of technologies designed to preserve parathyroid function further reinforces this concept. Near-infrared autofluorescence, indocyanine green angiography, and other adjunctive techniques offer promising opportunities to reduce parathyroid injury, yet their implementation remains heterogeneous, particularly within publicly funded healthcare systems [23,24,25]. Their clinical value can only be adequately evaluated if the complication they are intended to prevent is measured reliably. In this context, accurate quantification of p-HypoPT is a prerequisite for evidence-based investment in surgical innovation [26]. As previously observed for intraoperative neuromonitoring, widespread adoption of new technologies is facilitated when the burden of the complication they are designed to prevent is clearly recognised [27].

Permanent postoperative hypoparathyroidism is increasingly considered an important functional outcome in the assessment of thyroid surgery [6,7]. However, a standardised definition alone is insufficient for meaningful quality assessment if outcome ascertainment and clinical context remain heterogeneous. Reported rates may be influenced by case mix, indication and extent of surgery, duration and structure of follow-up, and centre- and surgeon-related factors. Crude percentages should therefore not be interpreted as direct measures of surgical quality. Meaningful comparison requires standardised long-term outcome ascertainment combined with procedure-specific and risk-adjusted reporting. The present cohort does not provide the information required for centre- or surgeon-level benchmarking; rather, it highlights the methodological conditions that would be necessary for such comparisons to be valid [28,29,30]. The present findings also highlight an important gap in longitudinal thyroid cancer registries. Meaningful assessment of long-term functional morbidity will require systematic integration of granular surgical variables, including the indication and extent of neck dissection, parathyroid management, surgeon- and centre-level characteristics, and the use of adjunctive technologies. Several of these variables are not currently captured systematically within the ITCO dataset. Their prospective incorporation across participating centres could enable more accurate characterisation of the determinants of p-HypoPT in future analyses and facilitate the generation of more clinically meaningful, procedure-specific and risk-adjusted reference data.

Within the variables available in the present study, central neck dissection showed the strongest association with p-HypoPT, consistent with previous reports [16,17,31,32,33]. This association should not be interpreted as evidence of a direct causal effect, as central neck dissection may also reflect greater oncological burden, more extensive surgery, and other unmeasured centre- or surgeon-related factors. Nevertheless, the marked difference in procedure-specific rates between total thyroidectomy without central neck dissection and procedures including central neck dissection reinforces the limitations of reporting a single aggregate p-HypoPT rate across heterogeneous thyroidectomy populations. Future reference rates should therefore be procedure- and indication-specific and appropriately adjusted for differences in case mix and surgical complexity. Such estimates may also improve preoperative counselling by providing patients with risk information that more closely reflects the planned procedure and underlying disease [34].

Beyond its oncological implications, one of the most original observations of the present study concerns the ATA 2025 risk stratification [5]. Although developed to refine prediction of disease recurrence, the updated classification identified subgroups with different unadjusted frequencies of p-HypoPT, particularly within the conventional intermediate-risk category. Importantly, this association was attenuated after adjustment for central and lateral neck dissection. Oncological and surgical complexity, however, should not be regarded as independent constructs: more extensive disease may necessitate more extensive surgery, which may in turn increase the risk of parathyroid injury [1,18]. Adjustment for neck dissection therefore enables assessment of whether ATA category carries an association with p-HypoPT beyond that attributable to surgical extent, while potentially accounting for a component of the clinical pathway through which oncological burden translates into postoperative morbidity. No formal mediation analysis was performed, and causal pathways cannot be established from these data. Accordingly, these findings should be considered exploratory and hypothesis-generating and do not support an independent predictive role of ATA classification for postoperative hypoparathyroidism. More broadly, they support the development of dedicated models for functional risk stratification. Such models could provide individualised preoperative estimates by integrating patient characteristics, disease burden, and the planned extent of surgery and could subsequently be updated as operative and postoperative information becomes available [18,34]. This approach could complement oncological risk stratification and strengthen patient counselling and shared decision-making. Notably, response to therapy at 12 months was comparable between patients with and without p-HypoPT, underscoring that favourable oncological outcomes may coexist with persistent long-term functional morbidity [1].

The strengths of this study include the large consecutive endocrine follow-up cohort, the long duration of observation, and the systematic annual reassessment performed by the same specialist endocrine team irrespective of disease severity, oncological status, or postoperative complications. This longitudinal setting allowed postoperative parathyroid function and treatment requirements to be assessed well beyond the immediate postoperative period.

Several limitations should nevertheless be acknowledged. First, the retrospective design is inherently subject to missing data and residual confounding. Moreover, the cohort was derived from a specialist endocrine follow-up programme rather than from a population-based surgical denominator; therefore, the observed frequency of p-HypoPT should not be interpreted as the population incidence after DTC surgery. Because inclusion required sufficiently documented ascertainment of the primary outcome, some selection bias related to outcome availability cannot be excluded.

Second, thyroid surgery had been performed across different hospitals within the Rome metropolitan area. This factor increases the heterogeneity of the cohort but precluded assessment of centre- and surgeon-specific characteristics and several potentially relevant intraoperative variables, including surgical volume, laterality and indication for central neck dissection, incidental parathyroidectomy, parathyroid autotransplantation, and the use of adjunctive technologies for parathyroid identification or perfusion assessment. Consequently, the associations observed for surgical variables, particularly central neck dissection, should be interpreted within the variables available in the present dataset rather than as evidence of a causal effect.

Third, p-HypoPT was ascertained using a treatment-based registry definition. Serum calcium and phosphate were available during endocrine follow-up, and patients receiving chronic calcium supplementation for indications unrelated to postoperative parathyroid dysfunction were excluded; however, PTH measurements were not systematically available in the study database, and the specific long-term replacement regimen and treatment doses were not systematically available. Supplementation was progressively reduced when clinically appropriate, but a standardised protocol requiring complete treatment withdrawal was not routinely applied. Some degree of outcome misclassification therefore cannot be entirely excluded.

Finally, the analyses based on the 2025 ATA risk stratification system were retrospective and exploratory and should be considered hypothesis-generating pending validation in prospective multicentre cohorts.

In conclusion, permanent postoperative hypoparathyroidism represents a clinically important long-term burden after thyroid cancer surgery. Meaningful quality assessment will require standardised long-term outcome ascertainment combined with procedure-specific, risk-adjusted reporting that accounts for disease and surgical complexity. Their prospective incorporation would enable more accurate characterisation of the determinants of p-HypoPT in future studies.

## Figures and Tables

**Table 1:** Baseline characteristics of the endocrine follow-up cohort.

Variable	Overall (*N* = 518)	%
**Patient characteristics**		
Age, years (mean ± SD)	48.1 ± 13.9	
Male	147	28.4%
Female	371	71.6%
**Tumor characteristics**		
**Histological subtype**		
PTC, classic	280	54.1%
PTC, follicular variant	168	32.4%
PTC, aggressive variants *	24	4.6%
PTC, subtype not specified	19	3.7%
FTC, minimally invasive	17	3.3%
FTC, widely invasive	4	0.8%
Oncocytic thyroid carcinoma	6	1.2%
**Primary tumor size**		
≤10 mm	295	56.9%
>10–20 mm	149	28.8%
>20–40 mm	57	11.0%
>40 mm	11	2.1%
Unknown	6	1.2%
Extrathyroidal extension **		
No	386	74.5%
Yes	132	25.5%
**Lymph node status**		
Unknown	227	43.8%
N0	194	37.5%
Central compartment	55	10.6%
Ipsilateral lateral compartment	15	2.9%
Central + ipsilateral lateral	21	4.1%
Central + contralateral lateral	2	0.4%
Bilateral lateral compartment	1	0.2%
Central + bilateral lateral compartment	3	0.6%
**Surgical characteristics**		
Extent of thyroid surgery ***		
Total thyroidectomy	494	95.4%
Lobectomy	24	4.6%
**Central neck dissection**		
No	363	70.1%
Yes	154	29.7%
Unknown	1	0.2%
**Lateral neck dissection**		
No	470	90.7%
Yes	47	9.1%
Unknown	1	0.2%
**Surgical margins**		
R0	312	60.2%
R1	21	4.1%
Unknown	185	35.7%
**ATA risk stratification system (2015)**		
Low	317	61.2%
Intermediate	180	34.7%
High	21	4.1%
**Response to treatment at 12 months**		
Indeterminate	236	45.6%
Structural incomplete	22	4.2%
Biochemical incomplete	22	4.2%
Excellent	238	45.9%

Summary of histological subtypes, surgical characteristics, and treatment characteristics in the long-term endocrine follow-up cohort. Abbreviation: SD, standard deviation; PTC: Papillary Thyroid Carcinoma; * PTC-Aggressive variant: Solid Variant, Insular Variant; Columnar Cell Variant, Hobnail Variant, Diffuse Sclerosing Variant, Tall Cell Variant, Tall Cell Variant with Trabecular Pattern; N0, no regional lymph-node metastases; R0, microscopically margin-negative resection; R1, microscopic residual disease; ATA, American Thyroid Association. ** Microscopic and macroscopic extrathyroidal extension were grouped as present. *** Near-total thyroidectomy was grouped with total thyroidectomy. Missing or unavailable information is explicitly reported as “Unknown”. Response to treatment at 12 months was classified according to ATA dynamic risk stratification.

**Table 2:** Clinical characteristics according to permanent postoperative hypoparathyroidism.

Variable	No p-HypoPT (*N* = 458)	p-HypoPT (*N* = 60)	*p*-Value
**Patient characteristics**			
Age, years (mean ± SD)	48.2 ± 14.0	47.5 ± 13.8	0.738
**Sex**			0.170
Male	135 (29.5%)	12 (20.0%)	
Female	323 (70.5%)	48 (80.0%)	
**Tumor characteristics**			
**Histological subtype**			0.083
PTC, classic	239 (52.2%)	41 (68.3%)	
PTC, follicular variant	154 (33.6%)	14 (23.3%)	
PTC, aggressive variants *	21 (4.6%)	3 (5.0%)	
Other histologies **	44 (9.6%)	2 (3.3%)	
Primary tumor size, mm (median [IQR])	9.0 [5.0–15.0]	10.0 [6.0–20.0]	0.348
**Primary tumor size category**			0.109
≤10 mm	263 (57.4%)	32 (53.3%)	
>10–20 mm	136 (29.7%)	13 (21.7%)	
>20–40 mm	47 (10.3%)	10 (16.7%)	
>40 mm	8 (1.7%)	3 (5.0%)	
Unknown	4 (0.9%)	2 (3.3%)	
**Extrathyroidal extension**			0.156
No	346 (75.5%)	40 (66.7%)	
Yes	112 (24.5%)	20 (33.3%)	
**Pathological nodal involvement**			**0.005**
N0	169 (36.9%)	25 (41.7%)	
Central compartment only	46 (10.0%)	9 (15.0%)	
Lateral compartment involvement ‡	28 (6.1%)	14 (23.3%)	
Unknown	215 (46.9%)	12 (20.0%)	
**Surgical characteristics**			
**Extent of thyroid surgery**			0.096
Total thyroidectomy	434 (94.8%)	60 (100%)	
Lobectomy	24 (5.2%)	0 (0%)	
Central neck dissection ***			**<0.001**
No	337 (73.6%)	26 (43.3%)	
Yes	120 (26.2%)	34 (56.7%)	
Unknown	1 (0.2%)	0	
**Lateral neck dissection**			**0.001**
No	423 (92.4%)	47 (78.3%)	
Yes	34 (7.4%)	13 (21.7%)	
Unknown	1 (0.2%)	0	
**Surgical margins**			0.742
R0	272 (59.4%)	40 (66.7%)	
R1	18 (3.9%)	3 (5.0%)	
Unknown	168 (36.7%)	17 (28.3%)	
**Radioactive iodine therapy**			0.295
No	130 (28.4%)	22 (36.7%)	
Yes	311 (67.9%)	38 (63.3%)	
Unknown	17 (3.7%)	0	
**Response to therapy at 12 months**			0.802
Indeterminate	206 (45.0%)	30 (50.0%)	
Structural incomplete	19 (4.1%)	3 (5.0%)	
Biochemical incomplete	19 (4.1%)	3 (5.0%)	
Excellent	214 (46.7%)	24 (40.0%)	

Values are n (%) unless otherwise indicated. p-values were calculated using Fisher’s exact test for binary categorical variables, Pearson’s χ^2^ test for multi-category variables, Welch’s t-test for age, and the Mann–Whitney U test for continuous tumor size. Missing or unknown observations were reported descriptively but excluded from inferential comparisons. * PTC-Aggressive variant: Solid Variant, Insular Variant; Columnar Cell Variant, Hobnail Variant, Diffuse Sclerosing Variant, Tall Cell Variant, Tall Cell Variant with Trabecular Pattern. ** Other histologies include PTC subtype not specified, follicular thyroid carcinoma, and oncocytic thyroid carcinoma. Lateral compartment involvement includes ipsilateral and/or contralateral lateral neck metastases, with or without central compartment involvement. Abbreviations: SD, standard deviation; IQR, interquartile range; PTC, papillary thyroid carcinoma; p-HypoPT, permanent postoperative hypoparathyroidism; N0, no regional lymph-node metastases; R0, microscopically margin-negative resection; R1, microscopic residual disease. *** Central neck dissection status is reported for the entire cohort; among the 494 patients undergoing total thyroidectomy, CND status was known in 493 patients (342 without and 151 with recorded central neck dissection). ‡ Overall adjusted p value from likelihood-ratio test for ATA category.

**Table 3:** Univariable and multivariable logistic regression for permanent postoperative hypoparathyroidism.

Variable	Univariable OR (95% CI)	*p*-Value	Multivariable OR (95% CI)	*p*-Value
**Patient-related factors**				
Age (per years)	1.00 (0.98–1.02)	0.739	1.01 (0.99–1.03)	0.272
Female sex	1.67 (0.86–3.25)	0.129	1.93 (0.96–3.88)	0.067
**Disease-related factors**				
Primary tumor size (per 10 mm)	1.23 (0.97–1.56)	0.081	1.23 (0.95–1.59)	0.115
Extrathyroidal extension	1.54 (0.87–2.75)	0.140	1.03 (0.54–1.94)	0.934
**Pathological nodal involvement**				
Any nodal involvement vs. N0	2.10 (1.12–3.94)	0.021	—	—
Central compartment only vs. N0	1.32 (0.58–3.03)	0.508	—	—
Lateral compartment involvement vs. N0	3.38 (1.57–7.28)	**0.002**	—	—
**Surgery-related factors**				
Central neck dissection	3.67 (2.12–6.37)	**<0.001**	2.94 (1.55–5.57)	**<0.001**
Lateral neck dissection	3.44 (1.70–6.98)	**<0.001**	1.78 (0.77–4.11)	0.178

*Values are odds ratios (ORs) with 95% confidence intervals (95% CIs). Primary tumor size was modeled per 10-mm increase. Reference categories were male sex, no extrathyroidal extension, no central neck dissection, no lateral neck dissection, and N0 for nodal involvement. The primary multivariable model included age, sex, primary tumor size, extrathyroidal extension, central neck dissection, and lateral neck dissection and was performed as a complete-case analysis (n = 510; 58 p-HypoPT events). No missing values were imputed. Eight patients were excluded from the model because of missing primary tumor size (n = 6), central neck dissection status (n = 1), or lateral neck dissection status (n = 1). Pathological nodal involvement was evaluated in univariable analyses only because of the high proportion of unknown nodal status and overlap with surgical extent variables. There was no evidence of relevant multicollinearity (all variance inflation factors ≤ 1.29). Model discrimination was moderate (AUC = 0.688), with satisfactory calibration (Hosmer–Lemeshow p = 0.889; Brier score = 0.095). In a parsimonious sensitivity model including primary tumor size, central neck dissection, and lateral neck dissection (n = 510; 58 events), central neck dissection remained associated with p-HypoPT (adjusted OR 2.80, 95% CI 1.51–5.22; p = 0.001); tumor size (adjusted OR 1.17, 95% CI 0.91–1.50; p = 0.233) and lateral neck dissection (adjusted OR 1.69, 95% CI 0.75–3.80; p = 0.203) were not statistically significant*.

**Table 4:** Permanent postoperative hypoparathyroidism according to ATA risk stratification system 2015 and the updated ATA 2025 risk stratification.

Risk Category	Patients, n	p-HypoPT, n (%)	95% CI (%)	Unadjusted OR (95% CI)	*p* Value	Adjusted OR * (95% CI)	*p* Value
**ATA 2015 risk stratification**							
Low	317	29 (9.1)	6.4–12.8	Reference	—	Reference	—
Intermediate	180	28 (15.6)	11.0–21.6	1.83 (1.05–3.19)	0.033	1.04 (0.54–1.98)	0.916
High	21	3 (14.3)	5.0–34.6	1.66 (0.46–5.96)	0.441	1.01 (0.26–4.00)	0.988
Overall comparison					0.093 †		0.994 ‡
**ATA 2025 risk stratification**							
Low	317	29 (9.1)	6.4–12.8	Reference	—	Reference	—
Low-intermediate	109	11 (10.1)	5.7–17.2	1.11 (0.54–2.32)	0.771	0.81 (0.37–1.73)	0.579
**High-intermediate**	**71**	**17 (23.9)**	**15.5–35.0**	**3.13 (1.61–6.08)**	**<0.001**	**1.66 (0.68–4.03)**	**0.263**
High	21	3 (14.3)	5.0–34.6	1.66 (0.46–5.96)	0.441	1.19 (0.30–4.66)	0.802
Overall comparison					0.005 †		0.566 ‡

The refined ATA 2025 risk stratification system subdivides the conventional ATA 2015 intermediate-risk category into low-intermediate and high-intermediate risk groups according to the updated risk stratification criteria. Percentages are calculated within each ATA risk category; 95% CIs for proportions were calculated using the Wilson method. * Adjusted models included ATA risk category, central neck dissection, and lateral neck dissection; complete-case n = 516 (60 events). Low risk was the reference category. † Overall unadjusted p value from Pearson’s chi-square test. ‡ Overall adjusted p value from likelihood-ratio test for ATA category. Sensitivity analysis restricted to total thyroidectomy yielded consistent results for ATA 2025 (global adjusted p = 0.468). Abbreviations: ATA, American Thyroid Association; CI, confidence interval; OR, odds ratio; p-HypoPT, permanent postoperative hypoparathyroidism.

## Data Availability

The data that support the findings of this study are available from the corresponding author upon reasonable request. The data are not publicly available due to privacy and ethical restrictions.

## Supplementary Materials

The following supporting information can be downloaded at: https://www.mdpi.com/article/10.3390/medsci14060650/s1, Supplementary File S1: STROBE checklist. Table S1: Comparison of patients with and without ascertainable permanent postoperative hypoparathyroidism status; Figure S1: Flow-chart of patient selection and ascertainment of permanent postoperative hypoparathyroidism.
