# Supplementary material for: Beyond Surgical Follow-Up: Measuring the Long-Term Burden of Permanent Hypoparathyroidism After Thyroid Cancer Surgery
Source: Med Sci (Basel). 2026 Oct 9;14(6):650. doi: 10.3390/medsci14060650 (PMC13706862; doi:10.3390/medsci14060650)
Supplement: Supplementary file 1 [file medsci-14-00650-s001.zip › medsci-4570987-supplementary.pdf]

Supplementary File S1. STROBE Checklist

STROBE Statement – Checklist for Cohort Studies

Manuscript: "Beyond Surgical Follow-Up: Measuring the Long-Term Burden of Permanent Hypoparathyroidism After Thyroid Cancer Surgery"

Completed according to the STROBE reporting recommendations for cohort studies. Page references correspond to the submitted manuscript layout; section/table/figure references are included to facilitate verification if pagination changes during production.

Section	Item	STROBE reporting item (condensed)	Where reported in manuscript
Title and abstract	1a	State the observational study design in the title or abstract.	Abstract, pp. 3–4 (retrospective analysis); Methods, p. 6 (retrospective observational study).
Title and abstract	1b	Provide an informative, balanced abstract describing methods and principal findings.	Abstract, pp. 3–4.
Introduction	2	Explain the scientific background and rationale.	Introduction, pp. 5–6.
Introduction	3	State the specific study objectives and any prespecified hypotheses.	Introduction, p. 6, final paragraph.
Methods	4	Present the key elements of the study design early in the manuscript.	Methods – Study design and population, p. 6.
Methods	5	Describe setting, locations and relevant dates, including study period and follow-up.	Methods – Study design and population, p. 6 (Sapienza University of Rome; surgery January 2013–January 2022; ≥12 months postoperative follow-up).
Methods	6a	Describe eligibility criteria, participant selection/source and follow-up.	Methods – Study design and population, p. 6; Figure S1, p. 23.
Methods	6b	For matched studies, report matching criteria and numbers.	Not applicable (unmatched cohort).
Methods	7	Define outcomes, predictors/exposures, potential confounders and diagnostic criteria.	Methods – Data collection and Outcome measures, pp. 6–7.
Methods	8	Describe data sources and methods of measurement/assessment for variables of interest.	Methods – Study design and population, Data collection, and Outcome measures, pp. 6–7 (institutional endocrine follow-up database; clinical, biochemical and treatment data).
Methods	9	Describe approaches used to limit or address potential sources of bias.	Methods, p. 6 (consecutive eligible patients, predefined eligibility criteria, prospectively collected database); Discussion – Limitations, p. 12.
Methods	10	Explain how the study size was determined.	Methods, p. 6: all consecutive eligible patients in the predefined study period were considered; no sampling or matching was performed.
Methods	11	Explain how quantitative variables were handled, including categorizations where applicable.	Statistical analysis, p. 7; Table 1, pp. 25–26; Table 3, p. 29 (tumour size categories for descriptive analyses; tumour size modelled per 10-mm increase in regression).
Methods	12a	Describe statistical methods, including adjustment for confounding.	Statistical analysis, pp. 7–8; Table 3, p. 29.
Methods	12b	Describe subgroup/interaction analyses where applicable.	Statistical analysis, p. 7 (hypothesis-generating comparison across ATA 2015 and 2025 risk categories); Table 4, p. 30.
Methods	12c	Explain how missing data were handled.	Tables 1–2, pp. 25–28 (unknown/missing values reported); Table 3, p. 29 (complete-case multivariable analysis, n=493; nodal status excluded from multivariable modelling because of missingness and overlap with surgical variables).
Methods	12d	Explain how loss to follow-up was addressed, if applicable.	Methods, p. 6 (≥12 months follow-up required for eligibility); Figure S1, p. 23 (patients with incomplete/missing follow-up excluded).
Methods	12e	Describe sensitivity analyses, if performed.	Not applicable; no sensitivity analyses were performed.
Results	13a	Report participant numbers at each stage of the study.	Results – Study population, p. 8; Figure S1, p. 23 (736 assessed; 522 included in final analysis).

Section	Item	STROBE reporting item (condensed)	Where reported in manuscript
Results	13b	Give reasons for non-participation/exclusion at each stage.	Methods, p. 6 (exclusion criteria); Figure S1, p. 23 (incomplete/missing follow-up data).
Results	13c	Consider presenting participant flow in a diagram.	Figure S1, p. 23.
Results	14a	Report participant characteristics and relevant clinical/exposure variables.	Results, p. 8; Table 1, pp. 25–26; Table 2, pp. 27–28.
Results	14b	Report the number of participants with missing data for relevant variables.	Tables 1–2, pp. 25–28 (unknown/missing categories explicitly reported where applicable).
Results	14c	Summarize follow-up time.	Methods, pp. 6–7: all included patients had at least 12 months of postoperative observation; p-HypoPT and response to therapy were assessed at the 12-month time point or beyond, as defined.
Results	15	Report outcome events or summary measures over time.	Results, pp. 8–9 (60/522 patients, 11.5% p-HypoPT); Table 4, p. 30.
Results	16a	Report crude and adjusted estimates with precision and identify variables included in adjustment.	Results – Factors associated with p-HypoPT, p. 9; Table 3, p. 29 (univariable and adjusted ORs with 95% CIs).
Results	16b	Report category boundaries when continuous variables are categorized.	Table 1, pp. 25–26 (tumour size categories); Table 3, p. 29 (tumour size per 10-mm increase).
Results	16c	Where relevant, provide absolute-risk information alongside relative estimates.	Results, pp. 8–9 and Table 4, p. 30 (absolute event rates and percentages reported for the full cohort and ATA risk groups).
Results	17	Report other analyses, including subgroup or exploratory analyses.	Results – ATA risk stratification and p-HypoPT, p. 9; Figure 2, p. 22; Table 4, p. 30.
Discussion	18	Summarize key findings in relation to the study objectives.	Discussion, p. 10, opening paragraph.
Discussion	19	Discuss study limitations, including potential bias or imprecision.	Discussion, p. 12 (retrospective design, multicentre surgery, inability to assess surgeon-specific/intraoperative factors, exploratory ATA 2025 analysis).
Discussion	20	Provide a cautious overall interpretation in the context of objectives, limitations and existing evidence.	Discussion, pp. 9–13.
Discussion	21	Discuss generalisability/external validity.	Discussion, p. 12 (limitations of the study setting and need for external validation in prospective multicentre cohorts).
Other information	22	Report funding sources and the role of funders.	Back matter – Funding statement (submitted manuscript).

Note: N/A = not applicable. The checklist is intended as a reporting aid and does not constitute an assessment of methodological quality.

Table S1. Comparison of patients with and without ascertainable permanent postoperative hypoparathyroidism status.

Four FT-UMP cases were included among the 522 patients with ascertainable p-HypoPT status at this stage of the selection process and were subsequently excluded from the final DTC analytic cohort, resulting in a final cohort of 518 patients.

Data are presented as n (%) unless otherwise indicated. Comparisons were performed to explore potential selection bias related to ascertainment of the primary outcome. No statistically significant differences were observed between groups in age, sex, primary tumour size, extrathyroidal extension, extent of thyroid surgery, central or lateral neck dissection, or ATA 2015 risk stratification. Missing or unavailable values are reported as "Unknown". Abbreviations: SD, standard deviation; p-HypoPT, permanent postoperative hypoparathyroidism; ATA, American Thyroid Association.

Variable	p-HypoPT outcome ascertainable (N = 522)	p-HypoPT outcome not ascertainable (N = 214)	p-value
**Age, years (mean ± SD)**	48.1 ± 13.9	48.5 ± 15.4	0.721
**Sex**			0.465
Male	148 (28.4)	55 (25.7)	
Female	374 (71.6)	159 (74.3)	
**Primary tumor size**			0.949
≤10 mm	296 (56.7)	116 (54.2)	
>10–20 mm	150 (28.7)	63 (29.4)	
>20–40 mm	58 (11.1)	27 (12.6)	
>40 mm	12 (2.3)	6 (2.8)	
Unknown	6 (1.1)	2 (0.9)	
**Extrathyroidal extension**			0.167
No	390 (74.7)	151 (70.6)	
Yes	132 (25.3)	62 (29.0)	
Unknown	0 (0.0)	1 (0.5)	
**Extent of thyroid surgery**			0.545
Total thyroidectomy	498 (95.4)	207 (96.7)	
Lobectomy	24 (4.6)	7 (3.3)	
**Central neck dissection**			0.260
No	367 (70.3)	143 (66.8)	
Yes	154 (29.5)	69 (32.2)	
Unknown	1 (0.2)	2 (0.9)	

**Lateral neck dissection**			0.266
No	474 (90.8)	189 (88.3)	
Yes	47 (9.0)	23 (10.7)	
Unknown	1 (0.2)	2 (0.9)	
**ATA 2015 risk stratification**			0.586
Low	321 (61.5)	126 (58.9)	
Intermediate	180 (34.5)	76 (35.5)	
High	21 (4.0)	12 (5.6)	

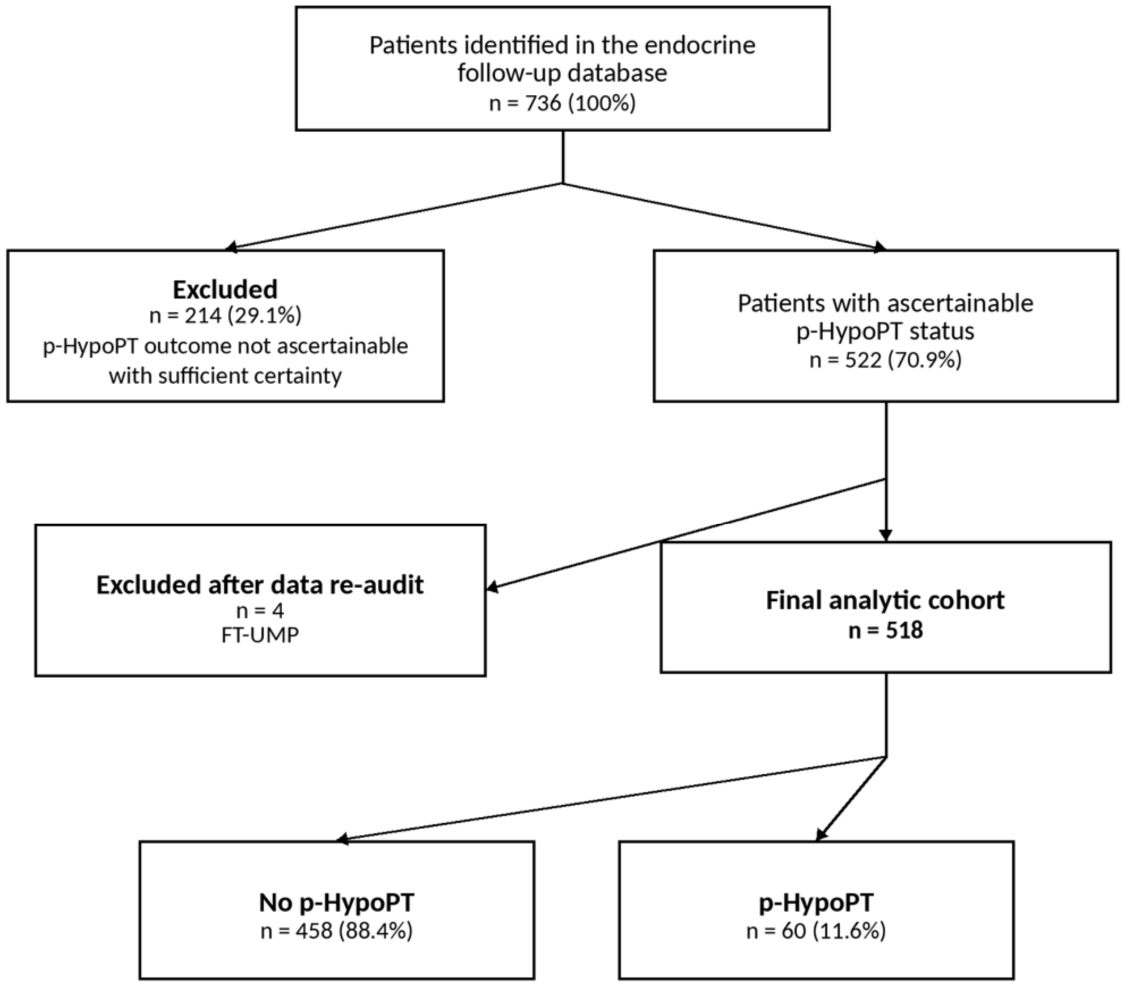

Supplementary Figure S1. Flow-chart of patient selection and ascertainment of permanent postoperative hypoparathyroidism.

Abbreviations: N: number; p-HypoPT: Permanent Hypoparathyroidism; FT-UMP: Follicular Tumor with Uncertain Malignant Potential.
